# Advanced brain aging in multiple system atrophy compared to Parkinson’s disease

**DOI:** 10.1016/j.nicl.2022.102997

**Published:** 2022-03-30

**Authors:** Chang-Le Chen, Ming-Che Kuo, Wen-Chau Wu, Yung-Chin Hsu, Ruey-Meei Wu, Wen-Yih Isaac Tseng

**Affiliations:** a Institute of Medical Device and Imaging, National Taiwan University College of Medicine, Taipei, Taiwan; b Department of Bioengineering, University of Pittsburgh, Pittsburgh, PA, USA; c National Taiwan University Cancer Center, Taipei, Taiwan; d Department of Neurology, National Taiwan University Hospital, National Taiwan University College of Medicine, Taipei, Taiwan; e Acroviz Inc., Taipei, Taiwan; f Molecular Imaging Center, National Taiwan University, Taipei, Taiwan

**Keywords:** Brain age, Magnetic resonance imaging, Multimodality, Multiple system atrophy, Parkinson’s disease, CT, cortical thickness, DSI, diffusion spectrum imaging, GFA, generalized fractional anisotropy, GM, gray matter, MD, mean diffusivity, MMSE, Mini-Mental State Examination, MoCA, Montreal Cognitive Assessment, MRI, magnetic resonance imaging, MSA, multiple system atrophy, PAD, predicted age difference, PD, Parkinson’s disease, ROI, region of interest, UMSARS, Unified Multiple System Atrophy Rating Scale, UPDRS, Unified Parkinson’s Disease Rating Scale, WM, white matter

## Abstract

Multiple system atrophy (MSA) and Parkinson’s disease (PD) belong to alpha-synucleinopathy, but they have very different clinical courses and prognoses. An imaging biomarker that can differentiate between the two diseases early in the disease course is desirable for appropriate treatment. Neuroimaging-based brain age paradigm provides an individualized marker to differentiate aberrant brain aging patterns in neurodegenerative diseases. In this study, patients with MSA (N = 23), PD (N = 33), and healthy controls (N = 34; HC) were recruited. A deep learning approach was used to estimate brain-predicted age difference (PAD) of gray matter (GM) and white matter (WM) based on image features extracted from T1-weighted and diffusion-weighted magnetic resonance images, respectively. Spatial normative models of image features were utilized to quantify neuroanatomical impairments in patients, which were then used to estimate the contributions of image features to brain age measures. For PAD of GM (GM-PAD), patients with MSA had significantly older brain age (9.33 years) than those with PD (0.75 years; P = 0.002) and HC (-1.47 years; P < 0.001), and no significant difference was found between PD and HC (P = 1.000). For PAD of WM (WM-PAD), it was significantly greater in MSA (9.27 years) than that in PD (1.90 years; P = 0.037) and HC (-0.74 years; P < 0.001); there was no significant difference between PD and HC (P = 0.087). The most salient image features that contributed to PAD in MSA and PD were different. For GM, they were the orbitofrontal regions and the cuneus in MSA and PD, respectively, and for WM, they were the central corpus callosum and the uncinate fasciculus in MSA and PD, respectively. Our results demonstrated that MSA revealed significantly greater PAD than PD, which might be related to markedly different neuroanatomical contributions to brain aging. The image features with distinct contributions to brain aging might be of value in the differential diagnosis of MSA and PD.

## Highlights

• MSA, but not PD, exhibits advanced brain aging in gray matter and white matter.
• Brain age of gray matter is correlated with that of white matter in PD.
• Brain age measures can partly reveal associations with symptom severity.
• Brain features underlying brain age difference between MSA and PD are identified.

## 1 Introduction

Multiple system atrophy (MSA) and Parkinson’s disease (PD) belong to alpha-synucleinopathy (Krismer and Wenning, 2017), but the two diseases manifest very different phenotypes (McCann et al., 2014). Compared to PD, patients with MSA exhibit poorer levodopa response to parkinsonism, additional cerebellar and autonomic deficits, and a shorter lifespan (Ozawa et al., 2004, Wenning et al., 2013). The trajectories of neurodegeneration also differ between MSA and PD; the pathology initially develops in the midbrain and ascends to the limbic system and association cortex in PD (Brooks and Tambasco, 2016), while greater and faster involvement in the cerebellum and striatum occurs in MSA (Brenneis et al., 2007). Early differential diagnosis of MSA and PD is desirable for appropriate treatment (Gilman et al., 2008). To date, however, a definite diagnosis can only be made based on autopsied alpha-synuclein (α-Syn) deposition in neurons as Lewy bodies in PD (Dufty et al., 2007) or in oligodendrocytes as glial cytoplasmic inclusions (GCIs) in MSA (Tu et al., 1998). Markers for early differentiation and detection of these two diseases are still lacking.

Magnetic resonance imaging (MRI) has been employed to capture macro- and microstructural alterations (Brooks and Tambasco, 2016, Schocke et al., 2002) in MSA and PD by targeting specific brain regions such as the substantia nigra, striatum, brainstem, and cerebellum (Krismer et al., 2019, Lewis et al., 2018) using various modalities (Barbagallo et al., 2016, Brooks and Tambasco, 2016, Schocke et al., 2002). Previous studies have identified higher regional apparent diffusion coefficients in the putamen in patients with the parkinsonian type of MSA than PD and healthy controls (HC) (Schocke et al., 2002), and atrophied putamen and infra-tentorial regions to be characteristic of MSA (Krismer et al., 2019). Hence, a concise measure of brain MRI characteristics is deemed to facilitate the differentiation between MSA and PD (Barbagallo et al., 2016, Brooks and Tambasco, 2016), which is not available yet.

Recent advance in the neuroimaging-based brain age paradigm provides a new window to reveal aberrant brain aging statuses in various brain disorders (Cole and Franke, 2017), which may serve as a biomarker of differential diagnosis. The established brain age model can be used to estimate brain-predicted age difference (PAD), the difference between an individual's brain-predicted and chronological age. Multiple studies have reported premature brain aging with elevated PAD in various brain disorders (Chen et al., 2019, Cole and Franke, 2017, Kaufmann et al., 2019). To date, studies of brain age on parkinsonism are scanty; only two neuroimaging studies reported equivocal or slightly elevated brain age in PD (Beheshti et al., 2020, Eickhoff et al., 2021). No research has yet been conducted to specifically investigate the variability of brain aging in alpha-synucleinopathies.

To evaluate whether the clinical discrepancy between MSA and PD could be detected by PAD measures, this study aimed to investigate the difference in brain aging between MSA and PD and to identify image features that underlie the aging variation. Given that PADs derived from different MRI modalities reflect different aspects of the brain aging status (Rokicki et al., 2021), and that MSA and PD have distinct neuropathological impairments in gray matter (GM) (Brooks and Tambasco, 2016) and white matter (WM) (Wenning et al., 2008), we hypothesized that brain age metrics derived from both GM and WM features and the neuroanatomical features attributing to brain aging were different between MSA and PD. In practice, we estimated modality-specific PADs and compared their differences between MSA and PD. We then investigated the image features that contributed to the resulting PADs in MSA and PD separately and identified the features with marked between-disease differences in brain aging contribution.

## 2 Materials and methods

### 2.1 Participants

**Fig. 1:**
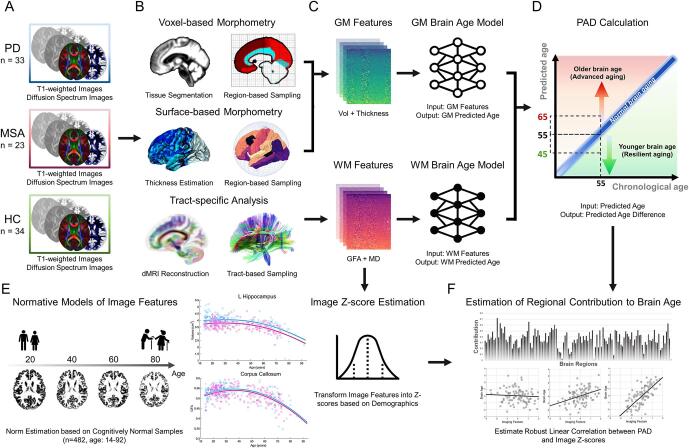
**The pipeline of image processing and brain age index estimation.** Structural and diffusion MRI data were acquired in each group (A). The structural MRI data were analyzed with voxel-based and surface-based morphometry, and the diffusion MRI data were processed with tract-based analysis (B). The quantified image features from each modality were used to estimate the brain age measures (C). Finally, the brain-predicted age difference (PAD) for gray matter (GM) and white matter (WM), representing brain age metrics, was used for further analyses (D). Besides brain age prediction modeling, spatial normative models of image features were established to quantify the structural deviance of brain regions for each participant (E). The estimated correlation magnitude between the structural deviance (Z-score) and the brain age measures (PAD) represents the strength of contribution to brain age for each image feature in each brain region (F).

Patients with MSA (N = 23; mean age = 64.6; standard deviation [SD] = 5.2; sex: 60.9% men) and PD (N = 33; mean age = 70.0; SD = 8.0; sex: 57.6% men) were recruited from the outpatient clinic of the Department of Neurology at National Taiwan University Hospital (NTUH) from 2019 to 2020 (Fig. 1A). Clinical diagnoses of MSA and PD were established by the consensus diagnostic criteria of *Gilman et al.* (Gilman et al., 2008) and the criteria of the United Kingdom Brain Bank (Hughes et al., 1993), respectively, and were conducted by experienced neurologists with more than 10 years of experience in this field (M.C. Kuo and R.M. Wu). ^99m^Tc-TRODAT-1 imaging was also used to confirm the clinical diagnosis (Chou et al., 2004, Lu et al., 2004). Patients with similar educational years and duration of disease were included, and those with psychiatric disorders, autoimmune disorders, major systemic diseases, or other known neurological diseases were excluded. The symptoms at initial recruitment were assessed using the Unified Parkinson’s Disease Rating Scale (UPDRS) and Hoehn and Yahr Scale for both diseases, and the Unified Multiple System Atrophy Rating Scale (UMSARS) for patients of MSA. The Montreal Cognitive Assessment (MoCA) and Mini-Mental State Examination (MMSE) were employed to measure cognitive abilities. We also enrolled 34 HCs (mean age = 65.1; SD = 5.6; sex: 52.9% men) who met the following inclusion criteria: the MMSE and MoCA score of ≥ 25, no self-reported substance abuse, no brain injury and surgery, no current serious health problem, and no history of neurological diseases or psychiatric disorders. The Institutional Review Board of NTUH approved the study (approval number: 201904092RINC), and all participants provided written informed consent.

For brain age modeling, brain MRI images of 482 cognitively normal participants (mean age = 36.9, SD = 19.1, range = 14–92; sex: 46.9% men) were obtained from the image database used in our previous studies (Chen et al., 2020a, Chen et al., 2019), including T1-weighted imaging and diffusion spectrum imaging (DSI) datasets; these images served as the training set for model building. Another independent set of images from 70 cognitively normal participants (mean age = 36.8, SD = 19.9, range = 14–83; sex: 47.8% men) from the database was used to test the reproducibility of the brain age models. These 552 participants met the aforementioned inclusion criteria for control participants and ethical approval.

### 2.2 MRI image acquisition

All brain images, including those from the training and independent test sets and those from patients with parkinsonism and their controls, were acquired using the same 3-Tesla MRI scanner (Tim Trio; Siemens, Erlangen, Germany) with a 32-channel phased-array head coil. High-resolution T1-weighted imaging was performed using a three-dimensional (3D) magnetization-prepared rapid gradient-echo sequence: repetition time/echo time (TR/TE) = 2000/3 ms, flip angle = 9°, field of view (FOV) = 256 × 192 × 208 mm^3, and isotropic spatial resolution = 1 mm^3. DSI was performed using a pulsed-gradient spin-echo echo-planar imaging sequence with a twice-refocused balanced echo: *b*_max_ = 4000 s/mm^2: TR/TE = 9600/130 ms, slice thickness = 2.5 mm, FOV = 200 × 200 mm^2, and in-plane spatial resolution = 2.5 × 2.5 mm^2. The diffusion-encoding acquisition scheme comprised 102 diffusion-encoding gradients corresponding to the Cartesian grids in the half-sphere of the 3D diffusion-encoding space (Kuo et al., 2008) and employed a bipolar diffusion-encoding gradient design to minimize the eddy current distortion artifact at the sequence level. Each MRI scan included T1-weighted imaging and DSI.

### 2.3 Image analysis

Image quality assurance (QA) was conducted before performing data analysis (Supplementary Material S1). All structural and diffusion MRI datasets used in this study passed the QA surveillance. For estimating brain age, we quantified GM and WM features to predict modality-specific brain age metrics (Fig. 1B & C). For GM features, voxel-based morphometry and surface-based morphometry were performed on T1-weighted images to quantify regional volume and cortical thickness, respectively, using the Computational Anatomy Toolbox (Gaser and Dahnke, 2016) (Fig. 1B). To estimate the volume of each region of interest (ROI), the LONI probabilistic brain atlas containing 56 ROIs (including cortical and subcortical regions) was used as a reference for volumetric tissue compartmentation (Shattuck et al., 2008). Surface-based morphometry was employed to measure cortical thickness through projection-based thickness estimation (Dahnke et al., 2013). The estimated thickness features were sampled according to the 68 cortical ROIs included in the Desikan–Killiany cortical atlas (Desikan et al., 2006). In sum, 56 volumetric features and 68 cortical thickness features were quantified.

To quantify WM features, we used an in-house algorithm called tract-based analysis (Chen et al., 2015). First, the diffusion indices, including generalized fractional anisotropy (GFA) and mean diffusivity (MD), derived from the DSI dataset were computed using the regularization version of the mean apparent propagator (MAP)-MRI framework (Ozarslan et al., 2013). To extract the effective features of WM, GFA and MD were sampled according to the spatial coordinates of 45 predefined major fiber tract bundles over the whole brain (Chen et al., 2015, Tung et al., 2021). After sampling the diffusion indices, we averaged each of the indices for each tract bundle, and 45 GFA and 45 MD features were obtained per participant to estimate WM-based brain age. The details of image processing are provided in Supplementary Material S2. Also, the parcellation of GM and WM ROIs is detailed in Supplementary Material S4.

### 2.4 Brain age modeling and estimation

GM-based and WM-based brain age prediction models were established using the training set’s GM and WM features, constituting 124 and 90 features, respectively (Fig. 1D). The sex factor was also included as a predictor in the models. We employed a 12-layer feed-forward cascade neural network architecture to predict brain age (Chen et al., 2020a). The loss function of model optimization was specified as a mean square error function. A 10-fold cross-validation procedure was adopted within the training set to estimate model performance. The training procedure was implemented using MATLAB R2019a (MathWorks Inc., Natick, MA, USA) with an NVIDIA GeForce RTX 2080Ti (NVIDIA Inc., Santa Clara, CA, USA) graphics processing unit for accelerated computing. The trained models were also applied to the test set for the evaluation of reproducibility. To quantify model performance, Pearson’s correlation coefficient and mean absolute error (MAE) between predicted age and chronological age were calculated. After the model performance was determined, we estimated the GM-based and WM-based PAD scores in MSA, PD, and HC groups for further analyses. Notably, given that the PAD directly derived from brain age models may have age-related bias according to previous reports (Smith et al., 2019), we used a well-established linear correction method proposed by *Cole et al.* to minimize the bias (de Lange and Cole, 2020).

### 2.5 Spatial normative modeling for image features

To estimate regional contributions of image features to brain age, we estimated the magnitude of regional alteration for each brain area and tract bundle through spatial normative modeling (Fig. 1E). The spatial normative modeling applied to neuroimaging features of a large-scale cognitively normal population-based cohort defines a normative range of neurobiological idiosyncrasies such as GM volume and WM microstructure, providing personalized statistical inferences useful for parsing the heterogeneity in clinical cohorts (Verdi et al., 2021a, Verdi et al., 2021b). Specifically, we employed the Gaussian process regression to establish spatial normative models for each neuroimaging feature at each ROI using the same training set for brain age modeling (Chen et al., 2019). Based on a cognitively normal population-based cohort given a certain age and sex, the normative models defined a statistical norm for each structural feature (Tung et al., 2021). This method allows the quantification of structural deviance of an individual from the norm. This deviance was equivalent to a standardized score (Z-score) and served as a measure of the structural integrity for each brain region. After ROI-based normative models were established, the normative models were applied to patients with MSA and PD to calculate the Z-score for each brain region. These estimated Z-scores were further used to calculate regional contributions to brain age estimates (i.e. PAD) in patients with MSA and PD. The details of spatial normative modeling are provided in Supplementary Material S3.

### 2.6 Statistical analysis

First, we compared PAD scores among MSA, PD, and HC groups. The PAD scores derived from GM and WM were compared using analysis of covariance while adjusting chronological age, sex, and education. *Post-hoc* analysis was used to test the difference between groups. The Benjamini–Hochberg method was used to address the multiple comparison problem. Partial correlation was employed to examine the correlation between GM-PAD and WM-PAD scores in each group while adjusting chronological age, sex, and education. In addition, associations of PAD measures with multiple clinical variables such as symptom severity and cognitive outcomes in MSA and PD were investigated. Moreover, we calculated feature importance to identify which regions contributed the most to brain aging in patients (Fig. 1F). This analysis consisted of two steps: (1) calculating patients’ Z-score profiles of structural features using ROI-based spatial normative models and (2) estimating the dependency between patients’ Z-score values and their PAD scores by using robust correlation estimation with MM-estimates (Salibian-Barrera, 2006). In step (1), we estimated the degree of deviance of brain features with respect to the normal range in terms of the Z-score, which indicated the extent of region impairment independent of age and sex. In step (2), we evaluated the association between the Z-score of an image feature and PAD. The resulting coefficient indicated the effect size of the feature association, representing the strength of contribution to the PAD score. The strength can reflect feature importance related to brain age in patients with parkinsonism, allowing us to investigate the difference in brain age contribution between MSA and PD.

## 3 Results

### 3.1 Characteristics of study participants

As shown in Table 1, patients with PD were chronologically older than patients with MSA and HC. The two patient groups did not exhibit significant differences in sex, education, disease duration, UPDRS Part I, and MMSE scores. However, patients with MSA had significantly earlier age of disease onset, worse motor performance in the Hoehn and Yahr stage, higher UPDRS Part II & Part III, and total scores, and lower performance in MoCA than patients with PD.

**Table 1:** Clinicodemographic characteristics of participants in each group.

Characteristics	Patients with MSA	Patients with PD	Healthy Controls	P-values
N	23	33	34	–
Age (y)	64.6 (5.2)	70.0 (8.0)	65.1 (5.6)	**0.003** (MSA vs. PD: **0.007**) (MSA vs. HC: 0.715) (PD vs. HC: **0.006**)
Sex (%)	60.9% men	57.6% men	52.9% men	0.832
Education (y)	10.6 (4.2)	11.1 (4.8)	15.0 (4.2)	**<0.001** (MSA vs. PD: 0.701) (MSA vs. HC: **<0.001**) (PD vs. HC: **0.001**)
Age at onset (y)	61.1 (5.2)	65.7 (8.9)	–	**0.031**
Disease duration (y)	3.5 (2.1)	4.7 (3.1)	–	0.114
MMSE	26.8 [9,30]	27.6 [22,30]	29.4 [28,30]	**0.026** (MSA vs. PD: 0.369) (MSA vs. HC: **0.034**) (PD vs. HC: **0.001**)
MoCA	20.1 [8,29]	25.1 [16,30]	28.3 [25,30]	**<0.001** (MSA vs. PD: **<0.001**) (MSA vs. HC: **<0.001**) (PD vs. HC: **0.013**)
Subtype	MSA-P: 7 MSA-C: 16	–	–	–
Hoehn & Yahr stage	3.4 (1.0) [1,5]	1.7 (0.7) [1,3]	–	**<0.001**
UPDRS I	2.5 (1.8) [0,6]	2.3 (1.7) [0,6]	–	0.686
UPDRS II	17.4 (8.5) [1,34]	5.4 (4.2) [1,17]	–	**<0.001**
UPDRS III	26.3 (16.3) [2,63]	9.7 (5.9) [1,28]	–	**<0.001**
UPDRS total	46.2 (24.4) [4,103]	17.4 (9.9) [2,46]	–	**<0.001**
UMSARS I	19.5 (8.2) [3,36]	–	–	–
UMSARS II	20.5 (9.8) [1,37]	–	–	–
UMSARS total	40.0 (16.8) [6,73]	–	–	–

Note:

**Bold** type: with significant difference over groups. ANOVA was used for the comparison of age and education across groups. ANCOVA (adjusting for age and education level) was used for the comparison of MMSE and MoCA across groups. The Chi-squared test was used for the comparison of sex across groups. Two-sample *t* tests were used for the comparison of the rest clinical variables (except UMSARS) between MSA and PD. Abbreviations: MMSE, Mini-Mental State Examination; MoCA, Montreal Cognitive Assessment; MSA, multiple system atrophy; MSA-C, cerebellar subtype; MSA-P, parkinsonian subtype; PD, Parkinson’s disease; UMSARS, Unified Multiple System Atrophy Rating Scale; UPDRS, Unified Parkinson’s Disease Rating Scale.

### 3.2 Comparison of PAD among MSA, PD and HC

**Fig. 2:**
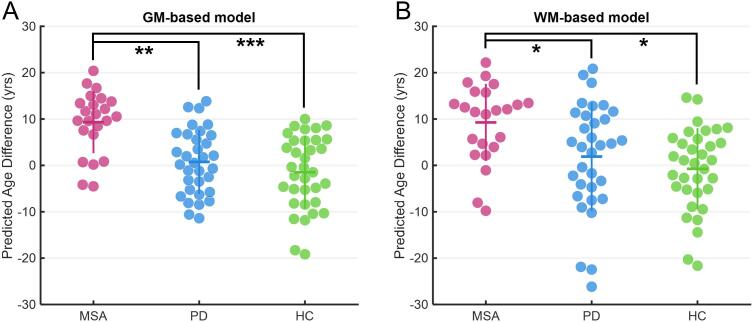
**Comparison of modality-specific PADs among PD, MSA, and HC.** One, two, and three asterisks denote the significant level at P < 0.05, P < 0.01, and P < 0.001, respectively.

The brain age models used in this study achieved satisfactory performance in the training and test sets (Supplementary Material S5). In the brain age analysis, although the individual variation of PAD was relatively high in the clinical groups, the PAD measures in MSA were significantly greater than those in PD and HC. GM-PADs were 9.33 ± 6.7 years in MSA, 0.75 ± 6.9 years in PD, and −1.48 ± 7.9 years in HC (*F*_(2,84)_ = 11.61, P < 0.001; PD vs*.* HC: P = 0.532, PD vs*.* MSA: P = 0.002, HC vs*.* MSA: P < 0.001), indicating increased GM brain age in MSA relative to other groups (Fig. 2A). Similarly, WM-PADs were 9.27 ± 8.3 years in MSA, 1.90 ± 11.7 years in PD, and −0.74 ± 8.8 years in HC (*F*_(2,84)_ = 4.80, P = 0.011; PD vs*.* HC: P = 1.000, PD vs*.* MSA: P = 0.026, HC vs*.* MSA: P = 0.019), revealing premature WM aging in MSA compared with PD and HC (Fig. 2B). In the Fig. 2B, some data points in each group seemed to be outliers that deviated downward from the mean. To confirm the statistical inference of the WM-PAD comparison, we excluded those observations (the lowest two in MSA, three in PD, and two in HC) and performed the comparison again. The results showed that WM-PADs were 11.01 ± 6.3 years in MSA, 4.44 ± 8.8 years in PD, and 0.52 ± 7.3 years in HC (*F*_(2,77)_ = 6.73, P = 0.002; PD vs*.* HC: P = 0.087, PD vs*.* MSA: P = 0.037, HC vs*.* MSA: P < 0.001), confirming the inference of WM-PAD comparison. All P-values shown in this section were corrected for multiple comparisons.

In the partial correlation analysis of PAD measures, a significantly positive correlation was found between GM-PAD and WM-PAD in PD (ρ = 0.409, P = 0.025), whereas no significant correlation was identified in MSA (ρ = 0.074, P = 0.757) and HC (ρ = -0.013, P = 0.949). When comparing the difference between each pair of correlation coefficients, no significant difference was found (MSA vs. PD: P = 0.212; PD vs. HC: P = 0.081; MSA vs. HC: P = 0.763).

Besides the comparison of PAD measures between MSA and PD, we also tested the association of PAD with multiple clinical variables including symptom severity, duration of illness, age of disease onset, and general cognitive performance in MSA and PD. Briefly, the motor function assessed using Part III of UPDRS was positively correlated (ρ = 0.236, P = 0.042) with GM-PAD in MSA, and that assessed using the total score of UPDRS was positively correlated (ρ = 0.316, P = 0.036) with GM-PAD in PD. Also, the score of Part I of UMSARS was positively correlated (ρ = 0.601, P = 0.030) with WM-PAD in the MSA group. The detailed statistics and brief discussion are provided in Supplementary Material S6.

### 3.3 Regional contributions of image feature to PAD in MSA and PD

**Fig. 3:**
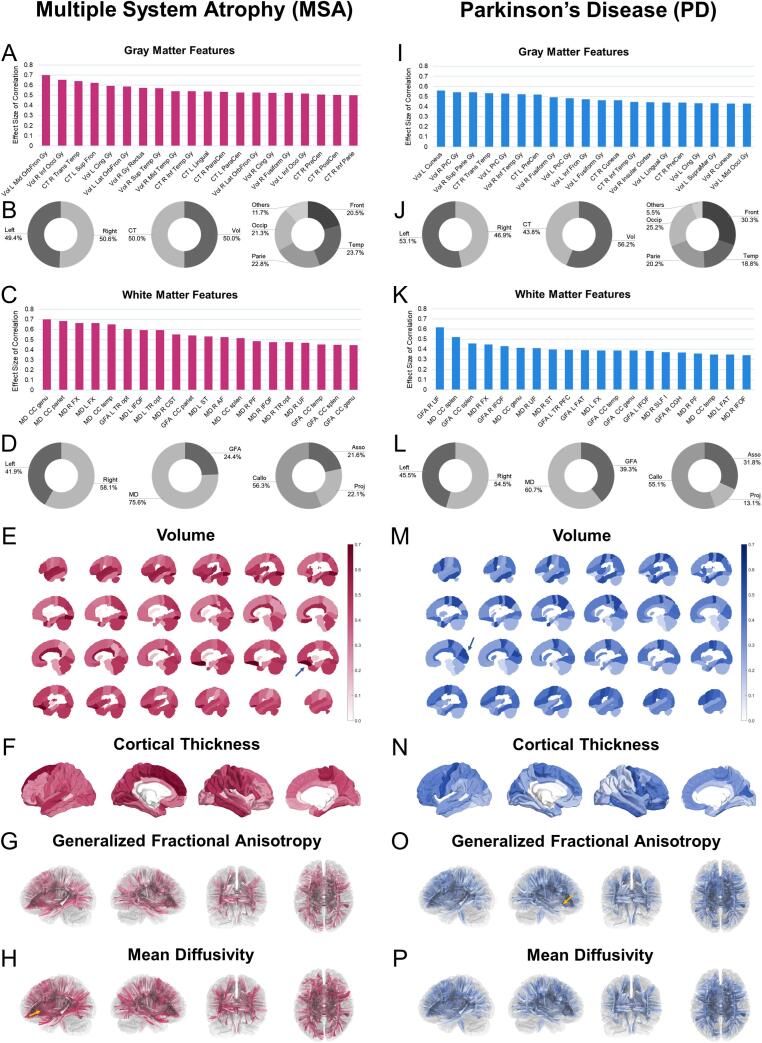
**Linear contributions of each brain region to PADs in MSA and PD based on different imaging modalities**. Higher effect sizes of correlation indicate stronger linear relationships between image features (Volume and CT for GM-PAD; MD and GFA for WM-PAD) and brain age metrics (A, C, I, and K). The percent ratio of regional contributions based on macroscopic parcellation is provided for each group and feature type (B, D, J, and L). The visualization of global contribution based on each image feature is shown in the bottom panel of this figure (E-H and M−P). The color spectrum in the brain maps indicates the coefficients of effect size. The arrows shown in the Panel E, H, M, and O indicate the most salient regions (i.e. the features with the highest effect size) of the gray matter and white matter in MSA and PD (left middle orbitofrontal gyrus in Panel E, corpus callosum of genu in Panel H, left cuneus in Panel M, and right uncinate fasciculus in Panel O). The code of labeling consists of feature type, hemisphere, and anatomical regions; for example, GFA R UF indicates the GFA of the right uncinate fasciculus. Abbreviation: Asso: association fiber system, Callo: callosal fiber system, CT: cortical thickness, Front: frontal lobe, GFA: generalized fractional anisotropy, MD: mean diffusivity, Occip: occipital lobe, Parie: parietal lobe, Proj: projection fiber system, Temp: temporal lobe, Vol: volume. The full name of neuroanatomical regions (A, C, I, and K) is provided in Supplementary Material S4.

We estimated the linear dependency between each image feature and PAD. The coefficient of linear dependency indicates the effect size of association; the higher the coefficient, the stronger the linear dependency with PAD. Fig. 3 shows the visualization of feature contributions to PAD and the top 20 features ranked as key ROIs in each patient group. In MSA, salient features contributing to GM-PAD included reduced cortical volumes in the left middle orbitofrontal gyrus, right inferior occipital gyrus, left cingulate gyrus, and left lateral orbitofrontal gyrus and decreased thickness in the right transverse temporal gyrus and left superior frontal gyrus (Fig. 3A). In PD, reduced volumes in the left cuneus, bilateral precentral gyri, and right superior parietal gyrus and thinner thickness in the right transverse temporal gyrus and left precentral gyrus were identified as strong contributors to GM brain aging (Fig. 3I). Among the identified features, the volumes in the right fusiform gyrus and left cingulate gyrus and the thickness in the right transverse temporal, inferior temporal, and precentral gyri were the features common to MSA and PD (Fig. 3A and 3I). In general, volume and cortical thickness had comparable contributions to brain age in MSA and PD (Fig. 3B and 3 J), and the contributing features did not show apparent inter-hemispheric lateralization (Fig. 3B and 3 J). As for the regional distribution, the frontal lobe appeared to contribute the most in PD, whereas there was no particular area with a leading contribution in MSA (Fig. 3B and 3 J). The brain mapping of contribution strengths (in terms of the effect size of linear correlation) was visualized in Fig. 3E, 3F, 3 M, and 3 N.

Regarding the features contributing to WM-PAD, the MD measures of the corpus callosum of the prefrontal lobe (genu), parietal lobe, and temporal lobe and the bilateral fornices had contribution strengths exceeding 0.6 in MSA (Fig. 3C). By contrast, in PD, only the GFA measures of the right uncinate fasciculus had an association strength larger than 0.6 (Fig. 3K). Generally, MD appeared to have stronger contributions than GFA in both MSA and PD (Fig. 3D and 3L), but MSA had much higher contribution strengths than did PD. Globally speaking, the contribution of the corpus callosum was most prominent in both MSA and PD, with slightly right hemispheric lateralization (Fig. 3D and 3L). The brain-wise mapping of contribution strengths was visualized in Fig. 3G, 3H, 3O, and 3P.

To observe the difference in contributing features between MSA and PD, we highlighted features that had an absolute difference of greater than 0.3 in coefficients (i.e. medium effect size difference) between MSA and PD. Several GM regions and WM tracts were identified to be distinct in their contributions to brain aging (Table 2). Most of the features had more weights in MSA than in PD whereas only GFA in the right uncinate fasciculus showed more weights in PD. GM features such as the left middle orbitofrontal gyrus, right lateral orbitofrontal gyrus, and right inferior parietal gyrus were more correlated with the GM-PAD changes in MSA compared to PD. On the other hand, WM features related to the thalamic radiations and corpus callosum were more associated with the WM-PAD of MSA. These selected features might potentially relate to the difference in brain age between MSA and PD.

**Table 2:** **GM and WM features with marked difference in brain age contribution (i.e. effect size of linear dependency) between MSA and PD.** The features were selected by having the difference in association effect size greater than 0.3 between MSA and PD. The strengths of brain age contribution in HC are provided as a reference. The abbreviation of each image feature is provided in Supplementary Material S4. ΔES = difference of effect size.

Measures	ROIs	MSA	PD	HC	ΔES_MSA-PD_
GM Vol	L Mid OrbFron Gy	0.700	0.220	0.353	0.479
	R Lat OrbFron Gy	0.527	0.106	0.496	0.421
	R Inf Occi Gy	0.652	0.325	0.577	0.327
	R Gy Rectus	0.574	0.254	0.290	0.320
GM CT	R Inf Parie Gy	0.501	0.068	0.365	0.433
	R PostCen Gy	0.502	0.103	0.519	0.400
	R SupraMar Gy	0.462	0.102	0.523	0.360
	L Insula	0.338	0.020	0.407	0.318
WM GFA	R UF	0.109	0.617	0.008	−0.507
	L TR Opt	0.604	0.261	0.358	0.343
	CC Parie	0.542	0.222	0.062	0.320
WM MD	L TR Opt	0.595	0.117	0.504	0.478
	CC Parie	0.683	0.277	0.502	0.406
	R ILF	0.444	0.041	0.143	0.403
	L TR SM	0.42	0.034	0.262	0.387
	L IFOF	0.595	0.225	0.427	0.370
	R TR Aud	0.427	0.076	0.340	0.351
	R TR Opt	0.475	0.129	0.392	0.346
	CC Temp	0.651	0.349	0.512	0.302

Abbreviation: CC: corpus callosum, CT: cortical thickness, GFA: generalized fractional anisotropy, GM: gray matter, Gy: gyrus, IFOF: inferior frontal occipital fasciculus, ILF: inferior longitudinal fasciculus, Inf: inferior, Lat: lateral, L: left, MD: mean diffusivity, Mid: middle, OrbFron: orbitofrontal, Parie: parietal lobe, PostCen: post central, R: right, SupraMar: supramarginal, Tem: temporal, TR Aud: thalamic radiation of auditory part, TR Opt: thalamic radiation of optic part, TR SM: thalamic radiation of sensorimotor cortex, UF: uncinate fasciculus, Vol: volume, WM: white matter.

## 4 Discussion

To our knowledge, this is the first study employing the brain age paradigm to investigate image idiosyncrasies in MSA and PD. We observed that patients with MSA had advanced brain age of 9.33 and 9.67 years in GM-PAD and WM-PAD, respectively. In contrast, patients with PD had relatively normal brain age (0.75 years in GM-PAD and 1.90 years in WM-PAD), underlining the feasibility of using brain age as imaging markers to distinguish the two diseases. Moreover, we expand the scope of brain age paradigm with normative modeling of image features, exploring the neuroanatomical alterations in MSA and PD. The findings could enrich the knowledge of neuropathological difference in MSA and PD behind the difference in brain age.

To date, only two studies investigate brain age in PD (Beheshti et al., 2020, Eickhoff et al., 2021). *Beheshti et al.* found that PD had GM-based brain age (average: +2.12 years) slightly higher than HC, but significantly lower than AD (average: +9.07 years). Another recent study also showed that GM-based brain age in PD was moderately higher (average: +2.9 years) than that in the normal cohort (Eickhoff et al., 2021). Both studies demonstrated that the advanced GM-based brain age in PD was mild to moderate as compared with HCs, which is consistent with our findings. Herein, our study extended similar findings to the WM-based brain age.

Unlike PD, advanced GM brain aging was found in MSA. In a longitudinal study, *Paviour et al.* reported that the atrophy rate of total or regional brain structures in PD was not significantly different from that in HC (Paviour et al., 2006), but patients with MSA showed faster atrophic changes, particularly in volumes of the midbrain, pons, and cerebellum than did patients with PD and HC. These findings imply that apparent GM-based brain aging may exist in MSA, but not in PD. Using the brain age paradigm, we found that GM-PAD was partly correlated with the severity of motor symptoms in both diseases. We also found that altered GM volume and cortical thickness in areas other than putamen, caudate, and cerebellum contributed strongly to the advanced GM brain aging in MSA. Therefore, we speculate that motor impairment might involve more extensive GM changes in MSA.

As for diffusion MRI, *Ji et al.* reported that MSA with the parkinsonian type had altered WM diffusivity in the bilateral corticospinal tracts and left anterior thalamic radiation as compared with PD and HC (Ji et al., 2015). Extensive WM abnormalities might reflect the pathological hallmark of GCIs in oligodendrocytes (Del Campo et al., 2021), which contribute to sophisticated functional deficits such as dysphagia (Clark et al., 2021), gait impairment (Chen et al., 2020b), and autonomic dysfunction (Snir et al., 2019) as shown in other diseases. These reports are in line with our findings in MSA that WM-PAD was positively correlated with functional impairment as graded by the UMSARS Part I scores. Interestingly, we demonstrated correlated GM-PAD and WM-PAD in PD but not in MSA or HC. This finding partly supports the prion-like spreading of alpha-synuclein in PD to initially unaffected cortexes and tracts through structural network connections (Taylor et al., 2018). Non-synchronization of GM and WM aging processes in MSA might reflect preferential WM involvement in its early stage.

Using spatial normative modeling, we quantified the structural deviance of image features and estimated the strength of each feature contributing to overall brain age metrics. From the results, we found markedly different contribution strengths between MSA and PD. Our results indicated that in the GM regions the orbitofrontal gyri presented markedly different contribution strengths between MSA and PD (Table 2). In fact, the atrophy of the orbitofrontal lobe in MSA was reported previously (Chang et al., 2009). *Lee et al.* used fluorodeoxyglucose positron emission tomography (FDG-PET) and identified multiple cortical regions with metabolism dysfunction, including the orbitofrontal area, as the hallmark of early MSA (Lee et al., 2008). Moreover, the reduced FDG uptake in the orbitofrontal lobe was correlated with disease severity and duration more significantly than in other dysfunctional regions (Lee et al., 2008). In a pathological–clinical correlation study, the deposition of GCI in the orbitofrontal cortex was correlated with disease severity more than that in other cortical regions (Brettschneider et al., 2018). Together, these studies support that the morphological changes in the orbitofrontal regions may be pivotal for driving the advanced GM aging in MSA.

The WM features with markedly different contributions to brain aging mainly involved the uncinate fasciculus, thalamic radiations, and corpus callosum (Table 2). Interestingly, these features predominantly showed higher contributions in MSA than PD, except for the right uncinate fasciculus which exhibited higher contributions in PD than MSA. The finding implies that the uncinate fasciculus might be more susceptible to neurodegeneration in PD. Consistently, the dysfunction of executive control was found to be the characteristic cognitive deficit in PD (Green et al., 2002), which was presumably related to the altered integrity of the uncinate fasciculus that interacted with the control systems in the prefrontal areas (Di Tella et al., 2020).

We found extensive WM tracts contributing to the advanced brain aging in MSA, especially those connecting cortical and subcortical regions. Our findings may help characterize tract alteration patterns in MSA (Chelban et al., 2019). In addition to the cerebellum and brainstem, previous studies have found extensive WM alterations in the superior corona radiata, body of the corpus callosum, and external and internal capsules (Péran et al., 2018). Alterations were also observed in motor fiber pathways including the left corona radiata to the bilateral posterior limbs of the internal capsule, cerebral peduncles, and bilateral middle cerebellar peduncles (Nguyen et al., 2021). These findings may reflect the pathological hallmark of MSA, i.e. the α-Syn-formed GCI is exclusively found in WM-related myelin-forming oligodendrocytes (Jellinger, 2018). Ishizawa et al. reported that GCI and microglial activation showed a higher correlation with demyelination in WM than with neuronal injury in GM (Ishizawa et al., 2008). The WM-restricted neuroinflammation may be a precipitating factor of the advanced WM aging in MSA (Hoffmann et al., 2019). Our findings of the extensive tract involvement in the WM brain aging in MSA may support the pathogenesis of MSA.

This study has limitations. First, the sample size was relatively small which might result in non-significant associations between brain age measures and image features, or overlapped variations in PAD between groups. Our results need to be validated by studies with larger cohorts. Second, the contributing features identified in our analyses might shift their strengths depending on the disease course, different prediction models, bias adjustment techniques, image processing, and brain atlases. For example, infratentorial structures such as the midbrain, pons, and cerebellum that are conventionally recognized as MSA-relevant brain regions were less weighted in our models. Whether these identified cerebral features could serve as biomarkers for differential diagnosis or prognosis remains to be explored. A longitudinal study is warranted to delineate their changes across disease stages. Finally, the patients with MSA had lower cognitive scores than did PD, which might be a confounding factor in our analysis. However, this concern is partially alleviated by our supplementary correlation analysis (Supplementary Material S6), which showed no significant correlations of PAD with MoCA and MMSE.

The present study explored the structural characteristics of brain aging status in MSA and PD, and obtained three novel findings. First, MSA, but not PD, exhibited advanced brain aging in both GM and WM compared to HC. Second, brain age measures can partly reveal the associations with symptom severity. Finally, we identified the neuroanatomical features underlying the brain age difference between MSA and PD. The most salient features included the orbitofrontal gyrus and anterior/central corpus callosum in MSA and the cuneus and uncinate fasciculus in PD. Given the unmet need of discriminating MSA from PD in the early stage, our results enrich the knowledge of neuropathological difference in MSA and PD, and might become potentially useful imaging biomarkers for early differential diagnosis of MSA and PD.

## Ethical approval

All procedures performed in this study involving human participants from the National Taiwan University Hospital (NTUH) were in accordance with the ethical standards of the NTUH Research Ethics Committee (REC) and with the 1964 Helsinki declaration and its later amendments or comparable ethical standards. Informed consent in the study was obtained from all individual participants who were recruited at the NTUH.

## Funding

This research was financially supported by (1) National Taiwan University and National Taiwan University Hospital (grant numbers: UN109-064) and (2) Ministry of Science and Technology of Taiwan (grant number: 106–2314-B-002–074-MY3 and 109–2314-B-002–120-MY3).

## CRediT authorship contribution statement

**Chang-Le Chen:** Conceptualization, Methodology, Software, Formal analysis, Investigation, Writing – original draft, Visualization, Writing – review & editing. **Ming-Che Kuo:** Conceptualization, Methodology, Investigation, Funding Acquisition, Writing – original draft, Writing – review & editing. **Wen-Chau Wu:** Investigation, Validation, Data Curation. **Yung-Chin Hsu:** Investigation, Validation, Data Curation. **Ruey-Meei Wu:** Validation, Supervision, Funding acquisition. **Wen-Yih Isaac Tseng:** Conceptualization, Supervision, Writing – review & editing, Project administration, Funding acquisition.

## Declaration of Competing Interest

The authors declare that they have no known competing financial interests or personal relationships that could have appeared to influence the work reported in this paper.
