# Supplementary material for: Advanced brain aging in multiple system atrophy compared to Parkinson’s disease
Source: Neuroimage Clin. 2022 Mar 30;34:102997. doi: 10.1016/j.nicl.2022.102997 (PMC8987993; doi:10.1016/j.nicl.2022.102997)
Supplement: Supplementary data 1 [file mmc1.docx]

**Supplementary Materials**

S1 Image Quality Assurance

S2 Image Data Processing

S3 Normative modeling for image features

S4 Abbreviations for Anatomical Regions in Gray Matter and White Matter

S5 Performance of Brain Age Prediction Model

S6 Association Analysis of Brain Age Measures with Clinical Variables

**S1 Image Quality Assurance**

Before we performed data analysis, all T1-weighted images underwent quality assurance (QA) procedures which are included in the Computational Anatomy Toolbox 12 (CAT12; http://dbm.neuro.uni-jena.de/cat.html), a retrospective QA framework for empirical quantification of quality differences. Retrospective QA involved automatic evaluation of essential image qualities such as noise, inhomogeneity, and image resolution. These quality measures were scaled to a rating scale, and “good” image quality level was required. Additional visual inspection was conducted to examine whether artifacts, including severe motion and abnormal lesions, remained in the images. All diffusion datasets also underwent QA procedures, including examinations for the signal-to-noise ratio (SNR), degree of alignment between T1- and diffusion-weighted images, and the motion-induced signal dropout (Chen et al., 2019). The SNR was evaluated by calculating the mean signal of an object divided by the standard deviation (SD) of the background noise (Dietrich et al., 2007). In practice, the signal was determined using a central square of an image for each slice, and the noise was averaged from 4 corner regions. Diffusion datasets with an SNR higher than mean SNR minus 2.5 SDs at their site were included. The degree of within-subject alignment between T1- and diffusion-weighted images was evaluated by calculating the spatial correlation between the T1-weighted image–derived WM tissue probability map and the diffusion-weighted image–derived generalized fractional anisotropy (GFA) map. Higher spatial correlation indicated greater spatial alignment between T1- and diffusion-weighted images. In addition, because of the relatively long scan time of DSI, in-scanner head motion would inevitably cause signal dropout in diffusion-weighted images, particularly in those with high *b* values. For this reason, all participants lay on the MRI table with the head packed with expandable foam cushions to restrict head movement. All acquired DSI datasets (5,712 images per participant) were examined by comparing the signal in the central square of each image with the predicted signal attenuation. Signal deviation from the predicted distribution was considered signal loss. Data with more than 60 images of signal dropout per participant (1% of the total diffusion-weighted images) were discarded. Notably, a prospective visual inspection was conducted to exclude datasets presenting severe head motion or unexpected brain lesions during scanning.

**S2 Image Data Processing**

In the image feature processing for GM, voxel-based morphometry and surface-based morphometry of 3D MPRAGE data were used. The image analyses were performed using an extension of the Statistical Parametric Mapping package (SPM12; https://www.fil.ion.ucl.ac.uk/spm/) (Ashburner et al., 2014) called Computational Anatomy Toolbox (CAT12; http://www.neuro.uni-jena.de/cat) (Gaser and Dahnke, 2016). For voxel-based morphometry analysis, we estimated regional gray matter volume by conducting the following procedures: (1) the T1-weighted images were spatially normalized and segmented into three major tissue classes (i.e. gray matter, white matter, and cerebrospinal fluid), (2) for spatial registration, the segmented tissue probability maps were taken and spatially registered onto the predefined standard templates by using the Geodesic Shooting algorithm (Ashburner and Friston, 2011), (3) the normalized maps were further modulated with the maps of Jacobian determinant to preserve the information of gray matter volumes of native space, and (4) the predefined LPBA40 probabilistic atlas containing 56 regions of interest (ROIs) (Shattuck et al., 2008) was transformed into the native space to estimate regional gray matter tissue volumes.

In addition, for surface-based morphometry analysis of cortical thickness, we applied the automated surface-preprocessing algorithms included in the CAT12 toolbox that enable the simultaneous estimation of cortical thickness of the left and right hemispheres by using the projection-based thickness method (Dahnke et al., 2013). Herein, cortical thickness was determined by estimating the WM distance based on tissue segmentation in the native space. The WM distance and a derived neighbor relationship were used to project local maxima (which is equal to the cortical thickness) onto other GM voxels. This approach included partial volume correction and correction for sulcal blurring and sulcal asymmetries. After the initial surface reconstruction, topological defects were repaired using spherical harmonics (Yotter et al., 2011a), and this topological correction was followed by a surface refinement, which resulted in the final central surface mesh. Subsequently, the individual cortical surface mesh was then reparameterized and spatially registered to the surfaced-based template using a spherical mapping with minimal distortions (Yotter et al., 2011b). After that, the Desikan–Killiany cortical atlas (containing 68 cortical ROIs) on the template was transformed to sample mean cortical features in the native space (Desikan et al., 2006). In this manner, 56 volumetric features and 68 cortical thickness features were obtained to estimate GM-based brain age and calculate the corresponding predicted age difference (PAD).

In the image processing for WM, our in-house algorithm called tract-based automatic analysis (Chen et al., 2015) was employed. First, the diffusion indices including GFA and mean diffusivity (MD) derived from the DSI dataset were computed using the regularization version of the framework of mean apparent propagator MRI (Hsu and Tseng, 2018; Ozarslan et al., 2013). The signal in 3D diffusion-encoding space was fitted with a series expansion of Hermite basis functions, which describe diffusion in various microstructural geometries (Avram et al., 2016). The zero-order term in the expansion series contained the diffusion tensor that characterizes the Gaussian displacement distribution. Higher-order terms in the expansion series were the orthogonal corrections to the Gaussian approximation, and these were used for reconstructing the average propagator. The MD in each voxel was determined by calculating the mean of the 3 eigenvalues of the diffusion tensor. We quantified GFA as the SD of the orientation distribution function divided by the root mean square of the orientation distribution function. To extract effective features of WM, the diffusion indices were sampled according to the spatial coordinates of 45 predefined major fiber tract bundles over the whole brain, which were constructed in the DSI template called NTU-DSI-122 (Hsu et al., 2015) through deterministic streamline-based tractography with multiple ROIs defined in the automated anatomical labeling atlas (Tzourio-Mazoyer et al., 2002). In practice, the sampling coordinates of major tract bundles were transformed from NTU-DSI-122 into individual DSI datasets with the corresponding deformation maps. The deformation maps were obtained through 2-step registration, which included anatomical information provided by the T1-weighted images and microstructural information provided by the DSI datasets (Hsu et al., 2012). The sampling coordinates were aligned with the proceeding direction of each fiber tract bundle, and diffusion indices were sampled in the native space along the sampling coordinates normalized and divided into 100 steps. Furthermore, we averaged the indices along 100 steps. Finally, 45 GFA features and 45 MD features were obtained for estimating WM-based brain age and calculating the corresponding PAD.

**S3 Normative modeling for image features**

Recent research has demonstrated that conceptualizing neurodegenerative diseases and mental disorders as deviations from normative functioning illustrates a new perspective to investigate the heterogeneous neurobiology underlying these disorders at an individual level (Marquand et al., 2019; Verdi et al., 2021a; Verdi et al., 2021b). The spatial normative modeling applied to neuroimaging features of a large-scale cognitively normal population-based cohort defines a normative range of neurobiological idiosyncrasies such as gray matter (GM) volume and white matter (WM) mesostructure, providing personalized statistical inferences and being useful for parsing the heterogeneity in clinical cohorts. This approach may also offer a new viewpoint when investigating the aberrant brain structures in neurological diseases such as Parkinson’s disease and multiple system atrophy. In this study, we leveraged the notion of normative modeling by establishing the statistical norm along with two demographical features (i.e. age and sex) and applied the spatial normative models to the clinical cohorts to quantify their individual deviance of neuroanatomical features. The individual deviance represented as Z-score profiles was used to correlate with the predicted age difference (PAD) so that we could estimate the regional contribution of image features to PAD, investigating potential key regions that had higher impacts on aberrant brain aging in the clinical patients.

To build spatial normative models, we used the training set (the same training set used for brain age modeling) to estimate the statistical norm of image features. In this study, we extracted 56 features for GM volume, 68 features for GM thickness, 45 features for WM generalized fractional anisotropy (GFA), and 45 features for WM mean diffusivity (MD). A single spatial normative model was fitted on each feature. Here, we employed Gaussian process regression (GPR), a flexible non-linear machine learning approach for regression, to obtain regression estimates of the normative model; the independent variables were chronological age and sex, and the dependent variable was a certain image features (e.g. left hippocampal volume). The GPR model estimated the mean and standard deviation (SD) of the training sample’s image feature given certain age and sex. In the model inference phase, an individual’s image feature was transformed to Z-score by the formula: $Zscore= \frac{feature - {\hat{\bar{x}}}_{peers}}{\hat{S}_{peers}}$, where ${\hat{\bar{x}}}_{peers}$ and $\hat{S}_{peers}$ were the estimated mean and SD of the image feature of the peers with the same age and sex derived from the GPR normative model, respectively. A higher/lower Z-score indicates a positive/negative deviance from the norm. The spatial normative model provides an individualized quantification of image feature, allowing us to evaluate individual abnormality of neuroanatomical idiosyncrasies. Here we provide a MATLAB pseudo code for the spatial normative modeling and its inference.

| % training  model_set = {};  for i = 1:num_features  % age, sex, and feature_set shown here belong to the training set  df = array2table([age,sex,feature_set(:,i)],’VariableNames’,{‘age’,’sex’,’feature’});  gpr_model = fit_gpr(df ,’feature ~ age + sex’);  model_set{i} = gpr_model;  end  % inference  z_score_profile = [];  for n = 1:num_subjects  for i = 1: num_features  % age, sex, and feature shown here belong to the test set (or target set)  gpr_model = model_set{i};  [mean_hat,std_hat] = gpr_model.predict(age(n),sex(n));  z_score = (feature(n,i) – mean_hat)/std_hat;  z_score_profile(n,i) = z_score;  end  end |
| --- |

**S4 Abbreviations for Anatomical Regions in Gray Matter and White Matter**

*Note: the anatomical parcellation was based on the following studies:*

| 1. Gray Matter Volume: Shattuck, David W., et al. "Construction of a 3D probabilistic atlas of human cortical structures." Neuroimage 39.3 (2008): 1064-1080. |
| --- |
| 1. Gray Matter Cortical Thickness: Desikan, Rahul S., et al. "An automated labeling system for subdividing the human cerebral cortex on MRI scans into gyral based regions of interest." Neuroimage 31.3 (2006): 968-980. |
| 1. White Matter Tract Bundles: Chen, Yu‐Jen, et al. "Automatic whole brain tract‐based analysis using predefined tracts in a diffusion spectrum imaging template and an accurate registration strategy." Human brain mapping 36.9 (2015): 3441-3458. |

**S5 Performance of Brain Age Prediction Model**

We performed 10-fold cross-validation on the training set, and the brain age models showed a strong linear correlation and low MAE (in the unit of year) between chronological age and brain predicted age based on GM features (ρ = 0.956, MAE = 4.34) and WM features (ρ = 0.944, MAE = 4.76). The models also accurately predicted brain age in the independent test set derived from GM features (ρ = 0.943, MAE = 4.69) and WM features (ρ = 0.967, MAE = 3.95). Given that the age-related bias in the PAD measures was minimized by using additional linear correction method (de Lange and Cole, 2020), the correlation analysis of the PAD scores against chronological age showed little age-related bias in PAD measures in both of the training and test sets (GM-PAD: ρ = -0.031, P = 0.495, and ρ = -0.096, P = 0.429, respectively; WM-PAD: ρ = -0.055, P = 0.226, and ρ = -0.006, P = 0.960, respectively). In the training set, the samples from younger age had relatively higher proportion than those from older age. To ensure that the brain age models would not have significantly larger prediction bias in the elderly that might confound our results, we split the training set into the older group (age >= 60 years) and the younger group (age < 60 years), and estimated the MAE in these two groups. The result showed that although a relatively higher MAE was observed in the older group (MAE = 5.09 in GM model and MAE = 5.11 in WM model) compared to the younger group (MAE = 4.52 in GM model and MAE = 4.62 in WM model), the difference of MAE was not significant between the groups (P = 0.184 for GM model and P = 0.302 for WM model). We conducted the same procedure in the test set and found a similar result; a relatively higher MAE was observed in the older group (MAE = 5.67 in GM model and MAE = 6.54 in WM model) compared to the younger group (MAE = 4.38 in GM model and MAE = 5.22 in WM model), the difference of MAE was not significant between the groups (P = 0.173 for GM model and P = 0.319 for WM model). These results confirmed that the brain age models did not yield significant prediction bias in the elderly.

**S6 Association Analysis of Brain Age Measures with Clinical Variables**

We performed a mass univariate regression analysis with a robust estimation (Salibian-Barrera, 2006; Salibian-Barrera and Zamar, 2002) to assess the linear relationship of clinical factors with PAD scores. The clinical factors consisted of symptom scores (i.e. UPDRS and UMSARS), clinical variables (i.e. duration of illness and onset age), and cognitive scores (i.e. MMSE and MoCA). The factors including chronological age, sex, and education were set as covariates dependent on empirically confounding effects. The multiple comparison problem was corrected using the Benjamini–Hochberg method.

In the association analysis, the motor function assessed using Part III of UPDRS was significantly correlated (P = 0.042) with GM-PAD in MSA, and that assessed using the total score of UPDRS was significantly correlated (P = 0.036) with GM-PAD in PD (Supplementary Table 1). Also, the score of Part I of UMSARS was significantly correlated (P = 0.030) with WM-PAD in the MSA group. Regarding cognitive measures, no significant correlation of PADs with MMSE or MoCA was identified. Regarding clinical variables, the age of onset and duration of illness were not correlated with either GM-PAD or WM-PAD in the two patient groups.

**Supplementary Table 1.** Associations of GM-PAD and WM-PAD with clinical factors in MSA and PD.

| **GM-PAD** | **MSA** | | |  | **PD** | | |
| --- | --- | --- | --- | --- | --- | --- | --- |
| Measures | Estimate | SE | P-value |  | Estimate | SE | P-value |
| UPDRS I | 1.162 | 0.930 | 0.454 |  | 1.598 | 0.775 | 0.096^-^ |
| UPDRS II | 0.321 | 0.183 | 0.096^-^ |  | 0.565 | 0.295 | 0.132 |
| UPDRS III | 0.236 | 0.094 | **0.042^*^** |  | 0.445 | 0.215 | 0.094^-^ |
| UPDRS total | 0.147 | 0.062 | 0.056^-^ |  | 0.316 | 0.126 | **0.036^*^** |
| UMSARS I | 0.314 | 0.189 | 0.114 |  | - | - | - |
| UMSARS II | 0.338 | 0.153 | 0.083^-^ |  | - | - | - |
| UMSARS total | 0.189 | 0.089 | 0.096^-^ |  | - | - | - |
| Duration of illness | 1.383 | 0.706 | 0.128 |  | 0.374 | 0.450 | 0.824 |
| Onset Age | -0.544 | 0.279 | 0.130 |  | -0.222 | 0.163 | 0.364 |
| MMSE | -0.385 | 0.396 | 0.690 |  | -0.696 | 0.976 | 0.482 |
| MoCA | -0.502 | 0.489 | 0.654 |  | -0.252 | 0.455 | 0.584 |
| **WM-PAD** | **MSA** | | |  | **PD** | | |
| Measures | Estimate | SE | P-value |  | Estimate | SE | P-value |
| UPDRS I | 1.185 | 1.240 | 0.351 |  | 0.619 | 1.035 | 0.554 |
| UPDRS II | 0.510 | 0.215 | 0.056^-^ |  | 0.173 | 0.404 | 0.672 |
| UPDRS III | 0.162 | 0.131 | 0.231 |  | 0.201 | 0.301 | 0.510 |
| UPDRS total | 0.136 | 0.082 | 0.111 |  | 0.117 | 0.172 | 0.500 |
| UMSARS I | 0.601 | 0.222 | **0.030^*^** |  | - | - | - |
| UMSARS II | 0.208 | 0.222 | 0.361 |  | - | - | - |
| UMSARS total | 0.214 | 0.121 | 0.094^-^ |  | - | - | - |
| Duration of illness | 0.643 | 0.951 | 0.507 |  | 0.069 | 0.578 | 0.906 |
| Onset Age | -0.084 | 0.387 | 0.830 |  | 0.004 | 0.208 | 0.986 |
| MMSE | -0.421 | 0.520 | 0.429 |  | 2.375 | 1.221 | 0.124 |
| MoCA | -0.451 | 0.670 | 0.515 |  | 0.766 | 0.567 | 0.376 |

Note:

1. adjusting age and sex for UPDRS and UMSARS series

2. adjusting sex for duration of illness and onset age

3. adjusting age, sex, and education for MMSE and MoCA

4. using Benjamini-Hochberg correction for multiple comparison problem

-: with marginal correlation

*: with significant correlation

**Brief discussion about correlations of motor and cognitive function with GM/WM brain aging**

Although patients with MSA exhibited advanced GM and WM brain aging, they only showed a few correlations with motor symptoms. WM-PAD was correlated with subjective symptoms in the UMSARS Part I score, and GM-PAD had a significant correlation with the UPDRS Part III score (Table 1). We postulated that motor-related regions only account for a small fraction of the weight in the brain age models in MSA. However, in PD, the significant correlation of GM-PAD with UPDRS total score may suggest a disease-specific effect of PAD on overall disease severity. Moreover, a trend of marginally positive correlation of PADs with both UPDRS and UMSARS in PD suggests that brain age metrics might reveal the association of motor symptom severity with a larger sample size. In our results, the brain age measures did not show any significant correlation with MMSE and MoCA in both MSA and PD groups. The use of classical cognitive rating tools (i.e. MMSE and MoCA) is less sensitive to executive dysfunction in our patients and might have led to decreased sensitivity to correlate with brain age. However, the lower mean MoCA score in patients with MSA comparing to PD partly reflects a more prominent executive dysfunction in MSA (Table 1). In fact, *Eschlböck et al.* reported that the Frontal Assessment Battery was more sensitive than MMSE score to detect executive dysfunction in 45 patients with a mean MMSE score of 27.6 points (Eschlböck et al., 2020). In another cohort of 372 patients with clinically possible or probably MSA, *Brown et al.* reported that higher percentages of patients exhibited impairment in executive function based on the Frontal Assessment Battery than the Dementia Rating Scale (30% vs. 20%) (Brown et al., 2010). Another possibility is that brain age measures might be preferentially associated with highly-deteriorated executive functions (Christman et al., 2020) or other higher-level cognitive processes, such as reward learning (Reyes et al., 2020) and decision-making (Rooks et al., 2019). Further studies are needed to investigate these hypothetical interpretations.

**Supplementary References**

Ashburner, J., Barnes, G., Chen, C.-C., Daunizeau, J., Flandin, G., Friston, K., Kiebel, S., Kilner, J., Litvak, V., Moran, R., 2014. SPM12 manual. Wellcome Trust Centre for Neuroimaging, London, UK 2464, 4.

Ashburner, J., Friston, K.J., 2011. Diffeomorphic registration using geodesic shooting and Gauss-Newton optimisation. NeuroImage 55, 954-967.

Avram, A.V., Sarlls, J.E., Barnett, A.S., Özarslan, E., Thomas, C., Irfanoglu, M.O., Hutchinson, E., Pierpaoli, C., Basser, P.J., 2016. Clinical feasibility of using mean apparent propagator (MAP) MRI to characterize brain tissue microstructure. NeuroImage 127, 422-434.

Brown, R.G., Lacomblez, L., Landwehrmeyer, B.G., Bak, T., Uttner, I., Dubois, B., Agid, Y., Ludolph, A., Bensimon, G., Payan, C., 2010. Cognitive impairment in patients with multiple system atrophy and progressive supranuclear palsy. Brain 133, 2382-2393.

Chen, C.L., Shih, Y.C., Liou, H.H., Hsu, Y.C., Lin, F.H., Tseng, W.I., 2019. Premature white matter aging in patients with right mesial temporal lobe epilepsy: A machine learning approach based on diffusion MRI data. Neuroimage Clin 24, 102033.

Chen, Y.J., Lo, Y.C., Hsu, Y.C., Fan, C.C., Hwang, T.J., Liu, C.M., Chien, Y.L., Hsieh, M.H., Liu, C.C., Hwu, H.G., Tseng, W.Y., 2015. Automatic whole brain tract-based analysis using predefined tracts in a diffusion spectrum imaging template and an accurate registration strategy. Hum Brain Mapp 36, 3441-3458.

Christman, S., Bermudez, C., Hao, L., Landman, B.A., Boyd, B., Albert, K., Woodward, N., Shokouhi, S., Vega, J., Andrews, P., 2020. Accelerated brain aging predicts impaired cognitive performance and greater disability in geriatric but not midlife adult depression. Translational Psychiatry 10, 1-11.

Dahnke, R., Yotter, R.A., Gaser, C., 2013. Cortical thickness and central surface estimation. NeuroImage 65, 336-348.

de Lange, A.G., Cole, J.H., 2020. Commentary: Correction procedures in brain-age prediction. Neuroimage Clin 26, 102229.

Desikan, R.S., Ségonne, F., Fischl, B., Quinn, B.T., Dickerson, B.C., Blacker, D., Buckner, R.L., Dale, A.M., Maguire, R.P., Hyman, B.T., 2006. An automated labeling system for subdividing the human cerebral cortex on MRI scans into gyral based regions of interest. NeuroImage 31, 968-980.

Dietrich, O., Raya, J.G., Reeder, S.B., Reiser, M.F., Schoenberg, S.O., 2007. Measurement of signal‐to‐noise ratios in MR images: influence of multichannel coils, parallel imaging, and reconstruction filters. Journal of Magnetic Resonance Imaging: An Official Journal of the International Society for Magnetic Resonance in Medicine 26, 375-385.

Eschlböck, S., Delazer, M., Krismer, F., Bodner, T., Fanciulli, A., Heim, B., Heras Garvin, A., Kaindlstorfer, C., Karner, E., Mair, K., 2020. Cognition in multiple system atrophy: a single‐center cohort study. Annals of clinical and translational neurology 7, 219-228.

Gaser, C., Dahnke, R., 2016. CAT-a computational anatomy toolbox for the analysis of structural MRI data. HBM 2016, 336-348.

Hsu, Y.C., Hsu, C.H., Tseng, W.Y., 2012. A large deformation diffeomorphic metric mapping solution for diffusion spectrum imaging datasets. Neuroimage 63, 818-834.

Hsu, Y.C., Lo, Y.C., Chen, Y.J., Wedeen, V.J., Isaac Tseng, W.Y., 2015. NTU-DSI-122: A diffusion spectrum imaging template with high anatomical matching to the ICBM-152 space. Hum Brain Mapp 36, 3528-3541.

Hsu, Y.C., Tseng, W.Y., 2018. An efficient regularization method for diffusion MAP-MRI estimation. 2018 ISMRM-ESMRMB Joint Annual Meeting.

Marquand, A.F., Kia, S.M., Zabihi, M., Wolfers, T., Buitelaar, J.K., Beckmann, C.F., 2019. Conceptualizing mental disorders as deviations from normative functioning. Molecular psychiatry 24, 1415-1424.

Ozarslan, E., Koay, C.G., Shepherd, T.M., Komlosh, M.E., Irfanoglu, M.O., Pierpaoli, C., Basser, P.J., 2013. Mean apparent propagator (MAP) MRI: a novel diffusion imaging method for mapping tissue microstructure. NeuroImage 78, 16-32.

Reyes, S., Rimkus, C.d.M., Lozoff, B., Biswal, B.B., Peirano, P., Algarin, C., 2020. Assessing cognitive control and the reward system in overweight young adults using sensitivity to incentives and white matter integrity. PloS one 15, e0233915.

Rooks, B., Anthony, M., Chen, Q., Lin, Y., Baran, T., Zhang, Z., Lichtenberg, P.A., Lin, F., 2019. A generic brain connectome map linked to different types of everyday decision-making in old age. Brain Structure and Function, 1-12.

Salibian-Barrera, M., 2006. Bootstrapping MM-estimators for linear regression with fixed designs. Statistics & probability letters 76, 1287-1297.

Salibian-Barrera, M., Zamar, R.H., 2002. Bootrapping robust estimates of regression. The Annals of Statistics 30, 556-582.

Shattuck, D.W., Mirza, M., Adisetiyo, V., Hojatkashani, C., Salamon, G., Narr, K.L., Poldrack, R.A., Bilder, R.M., Toga, A.W., 2008. Construction of a 3D probabilistic atlas of human cortical structures. NeuroImage 39, 1064-1080.

Tzourio-Mazoyer, N., Landeau, B., Papathanassiou, D., Crivello, F., Etard, O., Delcroix, N., Mazoyer, B., Joliot, M., 2002. Automated anatomical labeling of activations in SPM using a macroscopic anatomical parcellation of the MNI MRI single-subject brain. Neuroimage 15, 273-289.

Verdi, S., Kia, S.M., Marquand, A.F., Schott, J.M., Cole, J.H., Initiative, A.s.D.N., 2021a. The heterogeneous brain: Mapping individualised patterns of atrophy in Alzheimer’s disease using spatial normative models. Alzheimer's & Dementia 17, e057605.

Verdi, S., Marquand, A.F., Schott, J.M., Cole, J.H., 2021b. Beyond the average patient: how neuroimaging models can address heterogeneity in dementia. Brain 144, 2946-2953.

Yotter, R.A., Dahnke, R., Thompson, P.M., Gaser, C., 2011a. Topological correction of brain surface meshes using spherical harmonics. Human brain mapping 32, 1109-1124.

Yotter, R.A., Nenadic, I., Ziegler, G., Thompson, P.M., Gaser, C., 2011b. Local cortical surface complexity maps from spherical harmonic reconstructions. Neuroimage 56, 961-973.
